# The Receptor Tyrosine Kinase AXL in Cancer Progression

**DOI:** 10.3390/cancers8110103

**Published:** 2016-11-09

**Authors:** Erinn B. Rankin, Amato J. Giaccia

**Affiliations:** 1 Division of Radiation and Cancer Biology, Department of Radiation Oncology, Stanford University Medical Center, Stanford, CA 94305, USA; erankin@stanford.edu; 2 Department of Obstetrics and Gynecology, Stanford University Medical Center, Stanford, CA 94305, USA

**Keywords:** AXL, GAS6, metastasis, cancer stem cell phenotype, immune suppression, therapy, proliferation, resistance, small molecule, decoy receptor

## Abstract

The AXL receptor tyrosine kinase (AXL) has emerged as a promising therapeutic target for cancer therapy. Recent studies have revealed a central role of AXL signaling in tumor proliferation, survival, stem cell phenotype, metastasis, and resistance to cancer therapy. Moreover, AXL is expressed within cellular components of the tumor microenvironment where AXL signaling contributes to the immunosuppressive and protumorigenic phenotypes. A variety of AXL inhibitors have been developed and are efficacious in preclinical studies. These agents offer new opportunities for therapeutic intervention in the prevention and treatment of advanced disease. Here we review the literature that has illuminated the cellular and molecular mechanisms by which AXL signaling promotes tumor progression and we will discuss the therapeutic potential of AXL inhibition for cancer therapy.

## 1. Introduction

The AXL receptor tyrosine kinase (AXL) is a founding member of the TAM (TYRO3-AXL-MER) family of receptor tyrosine kinases (RTKs). The AXL gene was cloned from human chronic myelogenous leukemia (CML) cells where it encoded a 140 KDa protein with transforming ability [1]. The protein structure of AXL contains two immunoglobulin like domains (Ig) and two fibronectin III domains in the extracellular region, a single-pass trans-membrane domain, and an intracellular protein-tyrosine kinase domain [1]. The TYRO3 protein tyrosine kinase (TYRO3) and c-MER proto-oncogene tyrosine kinase (MERTK or MER) are structurally similar to AXL and contain two Ig and fibronectin III like domains in their extracellular domains [2,3]. There are two ligands that bind to and activate the TAM receptors including growth arrest-specific gene 6 (GAS6) and Protein S (PROS1) [4,5,6,7]. GAS6 and PROS1 are structurally similar and contain a sex hormone-binding globulin (SHBG) and gamma-carboxyglutamic acid (Gla) domains that mediate their activities. The SHBG domain binds to the Ig domains within the TAM receptors and mediates receptor dimerization and activation. While GAS6 can function as a ligand for all three TAM family receptors, GAS6 has higher binding affinity for AXL and TYRO3 over MER. In contrast, PROS1 binds to and activates TYRO3 and MER with little affinity for AXL (recently reviewed in [8]). Glutamic acid residues within the Gla domain are carboxylated in a vitamin K-dependent manner and bind to phosphatidylserine (PtdSer) residues that are exposed on activated platelets or apoptotic cells. Over the past 30 years, the TAM family of RTKs has emerged as an important factor controlling tissue homeostasis and innate immune responses. Additionally, dysregulation of TAM signaling has been associated with chronic inflammatory, autoimmune disease and cancer [8]. In this review we will highlight recent literature describing the role of GAS6/AXL signaling in cancer progression.

## 2. The Physiologic Role of GAS6/AXL Signaling

In contrast to most receptor tyrosine kinases, germline inactivation of the TAM receptors does not result in embryonic lethality. *Tyro3-Axl-Mertk* triple knockout mice are viable and fertile [9]. However, as these mice age, they develop a variety of degenerative diseases that are associated with the inability of phagocytes to clear apoptotic cells and membranes within adult reproductive, retina, and immune systems (for a recent review see [8]). Analysis of germline *Gas6* and *Axl* deficient mice indicates that GAS6/AXL signaling plays important roles in platelet aggregation and vessel integrity in the liver. Platelets from mice that are deficient for *Gas6*, or any one of the TAM receptors including *Axl*, are impaired in their ability to aggregate. While *Gas6*, *Axl*, *Tyro3*, or *Mertk* deficient mice do not suffer from bleeding under physiologic conditions, these mice are protected from life-threatening thrombosis. GAS6/TAM signaling on platelets activates PI3K/AKT signaling to stimulate tyrosine phosphorylation of the β3 integrin and amplify outside-in signaling via αIIbβ3 to promote platelet activation and aggregation [10,11]. In addition, both GAS6 and AXL are expressed by endothelial cells where they regulate vascular permeability in the liver [12].

## 3. GAS6 and AXL Expression in Cancer

Clinically, AXL is highly expressed in primary tumors and metastases in comparison to normal tissues. Immunohistochemical analysis of primary tumors revealed that AXL expression correlates with metastasis and/or poor survival in patients with lung adenocarcinoma, glioblastoma multiforme, pancreatic, renal cell carcinoma, esophageal adenocarcinoma, oral squamous carcinoma, pleural mesothelioma, ovarian adenocarcinoma, colon cancer, head and neck squamous cell carcinoma, urothelial carcinoma, esophageal cell carcinoma, and hepatocellular carcinoma (Table 1, [13,14,15,16,17,18,19,20,21,22,23,24,25,26,27,28]). Moreover, AXL expression correlates with drug resistance in patients with melanoma, myeloid leukemia, lung cancer, and renal cell carcinoma [29,30,31,32,33,34].

The majority of AXL signaling occurs in a ligand dependent manner mediated by GAS6. Activating mutations within the AXL kinase domain are rarely found in cancer (The Cancer Genome Atlas, TCGA). In cancer, AXL signaling can be activated by GAS6 in an autocrine or paracrine manner. Clinically, GAS6 expression in tumor specimens has been shown to be an adverse prognostic factor in urothelial, ovarian, lung adenocarcinoma, gastric cancer, and glioblastoma (Table 2, [14,23,31,35,36,37,38]). In addition, elevated serum GAS6 is an adverse prognostic biomarker in patients with oral squamous cell carcinoma and renal cell carcinoma [16,39]. Together, these studies implicate GAS6/AXL signaling as an important pathway driving tumor growth, metastasis, and drug resistance. Research over the past decade has focused on elucidating the functional role of GAS6/AXL signaling within the tumor microenvironment as well as determining the molecular mechanisms by which GAS6/AXL signaling promotes tumor progression. Most importantly, this work has led to the development of a variety of GAS6/AXL inhibitors that have been tested in preclinical and clinical studies and are promising new therapeutic strategies for cancer therapy.

## 4. Mechanisms of AXL Regulation in Cancer

**Figure 1:**
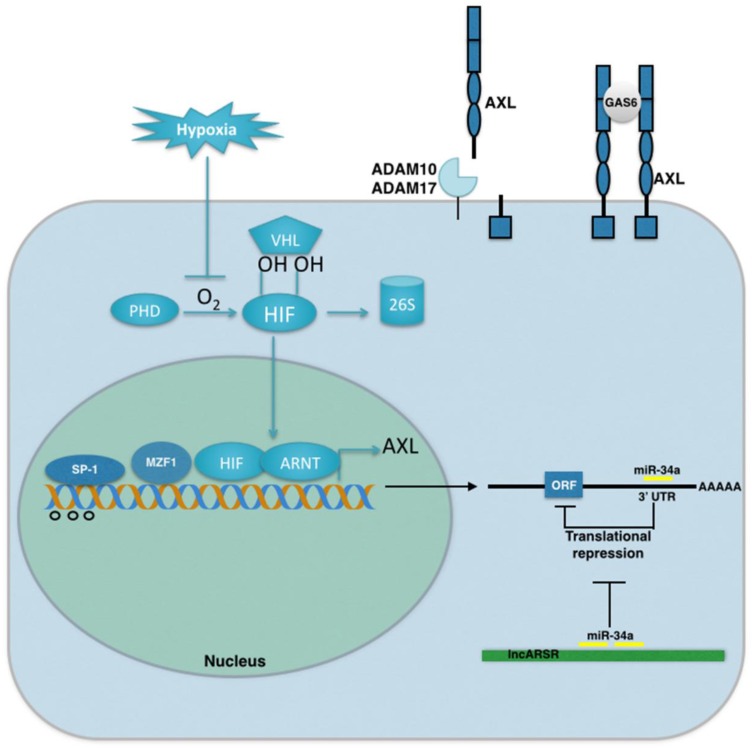
Mechanisms of AXL activation in cancer. AXL expression in cancer is regulated by transcriptional, translational, and posttranslational mechanisms. AXL transcription is regulated by promoter methylation (open circles) and the transcription factors: Specificity Protein Binding-1 and 3 (Sp-1, 3), Myeloid Zinc Finger 1 (MZF1), and the Hypoxia Inducible Factors HIF-1 and HIF-2. At the translational level, AXL is regulated by microRNA miR-34a. Finally, AXL is regulated by proteolytic shedding of the AXL extracellular domain cleaved by the metalloproteinases ADAM10 and ADAM17.

AXL signaling in cancer is regulated by genetic, epigenetic, and microenvironmental factors. Stress conditions within the tumor microenvironment play an important role in the activation of GAS6/AXL signaling. Hypoxia, or low oxygen tensions, are a prominent feature of solid tumors, associated with tumor progression, metastasis, and drug resistance [40]. In response to hypoxia, the hypoxia inducible factors HIF-1 and HIF-2 activate the expression of genes that mediate the cellular adaptive response to low oxygen tensions. AXL was recently identified as a direct transcriptional target of HIF-1 and HIF-2 in tumor cells where it mediates the prometastatic behavior of HIF signaling in von Hippel Lindau (VHL)-deficient renal cell carcinoma and hypoxic hepatocellular carcinoma cells [41]. Moreover, hypoxia inhibits GAS6-mediated downregulation of the AXL receptor in prostate cells [42]. Immunohistochemical staining of tumor tissues has shown that AXL, GAS6 and HIF-1 are coexpressed, supporting the concept that hypoxia and HIF activity is a mechanism for AXL upregulation in human cancers [42,43]. In addition to hypoxia, nutrient deprivation within the tumor microenvironment may also contribute to the activation of GAS6/AXL signaling. GAS6 was originally cloned as a factor that is abundantly expressed in serum starved NIH 3T3 cells and is negatively regulated after serum induction [44]. Functionally, GAS6 is sufficient to promote proliferation and inhibit apoptosis of serum starved NIH3T3 cells [45]. In addition to tumor cells, the vasculature, tumor-infiltrating leukocytes, and bone marrow progenitor cells are biologically relevant sources of GAS6 in the tumor microenvironment [14,46,47,48,49]. Together these findings highlight the important role of the tumor microenvironment in the activation of AXL signaling (Figure 1).

In addition to microenvironmental factors, AXL signaling can also be regulated by genetic and epigenetic mechanisms. The initial characterization of a 2.4 Kb fragment upstream of the translational start site within the AXL promoter revealed a minimal GC-rich region (−556 to +7) sufficient for basal Axl promoter activity. Within this region, two Sp-binding (specificity protein, Sp-1 and Sp-3), myeloid zinc finger (MZF1), and AP-1 (activator protein) binding sites regulate AXL promoter activity [50,51]. Moreover, methylated CpG sites have been identified within and around the Sp-binding sites indicating that promoter methylation may regulate Axl expression in cancer cells (Figure 1, [50]).

At the translational level, AXL is regulated by the microRNA, miR34a. MicroRNA-34a functions as a tumor suppressor in multiple tumor types by suppressing the expression of targets that control proliferation, apoptosis, and invasion [52]. A miR34a target site was identified within the AXL 3′-UTR where it binds and inhibits AXL expression in metastatic cancer cell lines [53,54,55]. In sunitinib-resistant renal cell carcinoma cells, the long non-coding RNA (lncRNA) lncARSR has recently been shown to sequester miR34a and facilitate AXL expression (Figure 1). Interestingly, lncARSR can be released by exosomes to transfer sunitinib resistance to neighboring cells. However, the role of AXL in the transfer of sunitinib resistance remains to be determined [56].

Finally, GAS6 binding to AXL is regulated at the posttranslational level through proteolytic shedding of the AXL extracellular domain (Figure 1). The ectodomains of many transmembrane substrates are cleaved by the metalloproteinases ADAM10 and ADAM17 that can mediate either positive or negative effects on signaling pathways. AXL was recently identified as a target of ADAM10 and ADAM17 where cleavage of the AXL ectodomain functioned as a negative regulator of endogenous AXL signaling [57]. Resistance to the MEK inhibitor, trametinib, was associated with decreased circulating levels of the AXL ectodomain in melanoma patients. MEK inhibition was shown to decrease AXL shedding on cancer cells by inhibiting ADAM10 catalytic activity through the activation of TIMP1, a negative regulator of ADAM10 [57]. These findings raise the intriguing possibility that the soluble AXL ectodomain may be useful as a diagnostic biomarker for clinical use. In support of this notion, soluble AXL receptor expression in blood has been found to be a negative prognostic factor in renal cell carcinoma, NF type 1 tumors, and hepatocellular carcinoma [16,58,59].

## 5. Mechanisms of AXL-Mediated Tumor Progression and Metastasis

**Figure 2:**
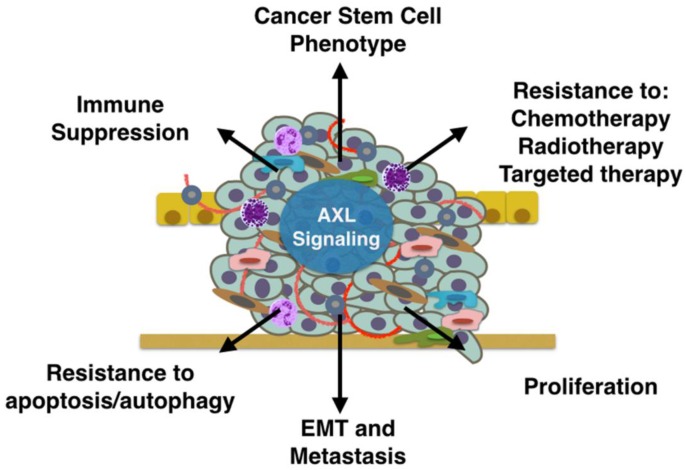
AXL signaling in the tumor microenvironment promotes tumor progression through multiple mechanisms. AXL signaling in tumor and stromal cells can promote immune suppression, the cancer stem cell phenotype, resistance to therapy, proliferation, epithelial-to-mesenchymal transition (EMT), metastasis, and resistance to apoptosis.

GAS6/AXL signaling promotes tumor progression and metastasis through multiple mechanisms. Here we will highlight recent studies that have implicated AXL signaling in tumor proliferation, survival, anti-apoptosis, drug resistance, the stem cell phenotype, metastasis, and immune suppression (Figure 2).

### 5.1. Proliferation, Survival, Anti-Apoptosis

AXL was originally cloned as a factor with transforming ability from CML cells. In these studies, overexpression of AXL in NIH 3T3 cells was found to promote neoplastic transformation [1]. In addition, GAS6 stimulation was found to promote the proliferation and survival of serum starved NIH 3T3 cells [45]. Subsequent studies demonstrated that GAS6/AXL signaling could protect adenovirus type 5 early region 1A gene (E1A) transfectants from apoptosis induced by serum deprivation through the activation of AKT signaling [60,61]. Together, these studies indicated that GAS6/AXL signaling might play an important role in the regulation of tumor growth. Indeed, genetic and therapeutic inhibition of AXL is sufficient to decrease tumor growth in some tumor types including squamous cell carcinomas, Kaposi sarcomas, pancreatic adenocarcinomas, mesotheliomas, schwannomas, ocular melanomas, B-cell chronic lymphocytic leukemia, and glioblastomas [15,62,63,64,65,66,67]. In contrast, the primary growth of other tumors such as ovarian, renal cell carcinoma, and breast cancer cells is not affected by AXL inactivation [41,68]. The ability of AXL signaling to promote tumor growth has been associated with the activation of downstream proliferation and/or survival pathways including MAPK, PI3K/AKT, and FAK/Src/NFKB signaling [15,64,69].

### 5.2. Drug Resistance

Drug resistance is a significant challenge in the clinical management of cancer. There are a variety of therapeutic strategies to treat cancer including conventional chemotherapy, radiotherapy, and targeted therapies. For each of these therapies, it has been observed clinically that tumors develop innate and acquired resistance mechanisms ultimately leading to tumor progression and poor patient outcome. Understanding mechanisms of resistance is important in the development of novel combination therapies that will be effective in improving overall survival rates in patients with advanced cancer.

Studies over the past decade have revealed that AXL is a key factor upregulated by tumor cells to promote resistance to multiple anti-cancer strategies. AXL was first described as a factor that promoted chemotherapy resistance in multiple tumor types including AML, non-small cell lung cancer, triple negative breast cancer, esophageal, and ovarian cancer [30,70,71]. AXL overexpression within chemoresistant tumors promotes survival through the activation of the AKT and ERK1/2 signaling pathways and decreases apoptosis through the modulation of c-ABL [71]. In addition to chemotherapy, AXL also promotes resistance to conventional radiotherapy in head and neck cancers [22].

AXL also promotes resistance to targeted cancer therapies. Multiple groups have reported that AXL is upregulated in response to EGFR inhibition where AXL-mediated signaling promotes resistance to these agents [31,72,73]. Most importantly, genetic and therapeutic inhibition of AXL is sufficient to restore sensitivity to EGFR inhibition in preclinical models [31,73]. Moreover, AXL overexpression is sufficient to induce resistance to the EGFR inhibitor cetuximab in head and neck cancer cell models [73]. It is thought that AXL may mediate resistance to EGFR inhibitors through the association and transactivation of signaling by AXL and ErbB family members [74]. Indeed AXL dimerization and phosphorylation of EGFR leads to the activation of phospholipase C gamma and protein kinase C that results in the activation of mTOR signaling to promote resistance to PI3K inhibition in head and neck cancer [75].

AXL is also an important factor driving resistance to MAPK inhibitors in BRAF (V600) mutant melanoma. The majority of melanomas harbor BRAF (V600E) mutations leading to the constitutive activation of the MAPK signaling pathway. As a result, MAPK inhibitors are utilized in the treatment of melanoma. However, the majority of patient tumors eventually develop resistance to these agents. Recent advances in single-cell RNA sequencing have allowed for the identification of gene expression programs that are associated with rare drug-resistant populations within human melanoma tumors. These studies have revealed that MITF low and AXL high gene expression program correlates with MAPK inhibitor resistance in melanoma patients [29,32]. Moreover, an independent study utilized RNA-seq analysis to identify factors that protect MITF low melanoma cells from MAPK inhibition and identified AXL as a key RTK upregulated during resistance [32]. Importantly, inhibition of AXL with the R428 small molecule (Bergen Bio, Bergen, Norway) is sufficient to sensitize melanoma cells to MAPK inhibition, suggesting that AXL inhibition may be an effective strategy to prevent and inhibit MAPK resistance in patients with melanoma [33].

### 5.3. Stem Cell Phenotype

The tumor cell population within an individual tumor is heterogeneous with a rare population of cells that have the ability to renew and enhance tumor initiating potential and long-term repopulation potential. The cancer stem cell phenotype promotes resistance to anti-cancer therapies, metastasis, and tumor dormancy. Therefore, understanding the mechanisms that drive the cancer stem cell phenotype are important to develop therapeutic strategies to target this aggressive cell population [76].

Recent studies have implicated AXL as an important factor driving the cancer stem cell phenotype. In cutaneous squamous cell carcinoma cell lines, AXL expression correlates with the cancer stem cell markers CD44 and ALDH1, increased resistance to chemotherapy, and enhanced sphere formation [77]. Similarly, in breast cancer cells, AXL expression correlates with the stem cell markers Isl1, Cdc2a, and Bglap1. Treatment with an AXL inhibitor, MP470 (an AXL, PDGFRa, ckit inhibitor; SuperGen, Dublin, CA, USA) reduced the ability of breast cancer stem cells to form mammospheres and improved tumor cell sensitivity to chemotherapy [78]. In glioblastoma stem-like cells AXL inhibition reduced the self-renewal capacity in vitro and inhibited the growth of patient derived xenografts in vivo [79]. Collectively, these studies suggest that AXL may be a marker and functional driver of the cancer stem cell phenotype.

### 5.4. Metastasis

AXL expression appears to increase during cancer progression and metastasis. In preclinical studies, genetic inactivation of AXL is sufficient to decrease the migratory, invasive and metastatic potential of tumor cells [15,17,28,41,42,51,62,63,68,80,81]. While there are multiple mechanisms by which AXL signaling promotes metastasis, the majority of studies have focused on defining the role of GAS6/AXL signaling in the early stages of metastasis.

The early stages of metastasis begin with local tumor cell migration and invasion within the primary tumor environment into the local vasculature. This process has been associated with tumor epithelial-to-mesenchymal transition (EMT), which allows cells to detach from neighboring cells through the downregulation of cell-cell adhesion molecules such as E-cadherin. AXL is highly expressed by mesenchymal tumor cells where it plays an important role in maintaining the mesenchymal and invasive phenotype. Genetic inactivation of AXL in mesenchymal tumor cells leads to a decrease in the expression of mesenchymal markers, an increase in the expression of the epithelial marker E-cadherin, and reduced invasive potential [15,34,41,42,62]. While AXL mediated EMT may be an important mechanism by which tumors promote metastasis, the mechanisms by which AXL signaling promotes EMT remain poorly understood. Recent studies have indicated a role for lateral receptor crosstalk through signaling molecules such as Src family kinases (SFKs), the Elmo scaffold protein, AKT, NF-κB, and GSK3β in AXL mediated invasion [41,62,81]. Recent studies suggest that AXL may be both an upstream inducer and a downstream effector of the EMT phenotype. Induction of EMT through TGFβ results in the upregulation of AXL expression that is important in maintaining an EMT phenotype [28,70]. Notably, pharmacologic inhibition of AXL in mesenchymal tumor cells is sufficient to sensitize these cells to anti-mitotic agents [70].

Another important mechanism by which AXL signaling promotes tumor cell invasion and migration is through the activation of matrix metalloproteinases (MMPs). Tumor cells upregulate the expression and secretion of matrix metalloproteinases to breakdown the extracellular matrix and release growth factors to promote tumor invasion and growth [82]. AXL activation leads to enhanced invasion that is associated with increased expression and activity of MMPs [15,68,80].

### 5.5. Immune Suppression

A critical step in tumor progression is the ability of tumor cells to evade immune attack. Accumulating evidence suggests that AXL may promote an immunosuppressive phenotype within the tumor microenvironment.

Macrophages are a key cellular component of the tumor microenvironment that contributes to tumor progression and drug resistance. Clinically, the presence of macrophages within tumors is associated with poor patient survival in a variety of tumor types. Functionally, macrophages can promote tumor cell survival, angiogenesis, and promote T cell suppression [83]. An important physiological role for the TAM family members in the regulation of macrophage clearance of apoptotic cells and suppression of inflammatory immune responses has been previously described (for a recent review see [84]). Most notably, genetic inactivation of all three TAM family members results in the development of a severe lymphoproliferative disorder accompanied by broad-spectrum autoimmunity [85]. These phenotypes are associated with the inability of dendritic cells and macrophages to clear apoptotic cells, and a failure to downregulate innate inflammatory responses [86]. While AXL is highly expressed on tumor associated macrophages, the functional role of AXL in these cells remains poorly understood [87]. Systemic inhibition of AXL signaling in the tumor microenvironment with the treatment of an AXL monoclonal antibody inhibited inflammatory cytokine secretion from tumor associated macrophages in a breast cancer model [87]. Additionally, in vitro co-culture studies have indicated a role for tumor AXL signaling in M2 polarization of THP-1 cells [88]. Future studies elucidating the role of both tumor and macrophage AXL signaling are needed.

Natural killer (NK) cells are innate lymphoid cells that are important in controlling anti-tumor responses. Recent studies have demonstrated that NK cells have features of adaptive immunity as well through their antigen specificity and ability to mount long-livered memory responses. NK cells primarily function through the rapid release of inflammatory cytokines such as interferon gamma and can directly kill infected cells through the secretion of lytic granules. Within the bone marrow, NK cells differentiate and mature through a series of stages. Interestingly, NK cells are derived from a common lymphoid progenitor cell that develops into an NK cell precursor (NKP) and immature NK cells (iNK). As NK cells mature in the bone marrow (marked by the expression of CD11b, CD43, Ly49, CD49b), they gain functional competence in cytotoxicity and interferon gamma expression and egress from the bone marrow (for a review see [89]). Recent studies have suggested an important role for TAM signaling in NK cell differentiation and anti-tumor activity. TAM receptor inhibition with a novel small molecule TKI inhibitor (LCD1267; Lead Discovery Center GmbH, Dortmund, Germany) resulted in decreased metastasis associated with enhanced NK cell anti-tumor activities in the murine model of B16 melanoma. Adoptive transfer of NK cells treated with LCD1267 recapitulated antitumor responses, and NK cell depletion abolished the therapeutic benefits of the inhibitor, suggesting that the anti-tumor effects of this treatment were mediated at least in part through NK cell activity [90]. While these findings indicate an important role for TAM signaling in anti-tumor responses mediated by NK cells, future studies are needed to determine the specific and relative contributions of AXL, TYRO3, and MER to NK cell antitumor activities. This is particularly important, as a role for AXL and TAM signaling in NK cell development and effector function has been observed by multiple groups. In human NK cells, blockade of GAS6 signaling with a soluble AXL decoy receptor inhibited IL-15 mediated NK cell development from human CD34 hematopoietic progenitor cells [91]. Similarly, in murine NK cells AXL and TAM family receptors are essential for the generation of functional NK cells in mice. Genetic inactivation of AXL, or the entire TAM family can lead to a block in NK cell maturation in the bone marrow and inhibits the cytotoxic activity and effector function of NK cells in vitro [92]. It will be important in future studies to utilize specific or combination TAM deficient mice to determine the relative contributions of the TAM family in NK cell anti-tumor responses.

## 6. Therapeutic Targeting of AXL

**Figure 3:**
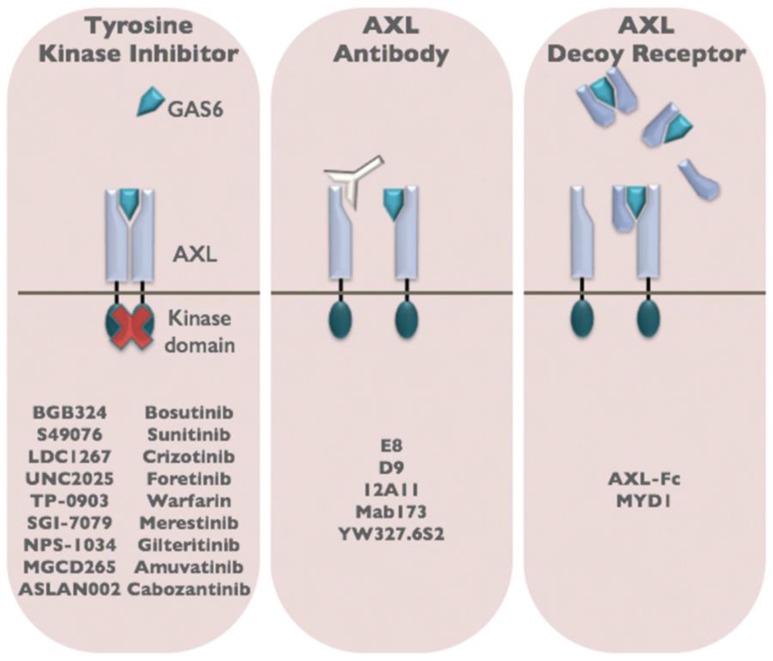
Three classes of AXL inhibitors have been developed for cancer therapy. Small molecule tyrosine kinase inhibitors that block AXL kinase activity, anti-AXL antibodies, and soluble AXL decoy receptors are in preclinical and clinical development for the treatment of cancer.

The clinical and preclinical studies described above indicate an important role for AXL in tumor progression, metastasis, and drug resistance. These findings raise the intriguing possibility that therapeutic targeting of AXL may be an effective anti-cancer strategy. AXL is an attractive therapeutic target because the majority of AXL signaling occurs in a ligand-dependent manner mediated by GAS6. In cancer, GAS6/AXL signaling can be activated in an autocrine or paracrine manner with tumor cells as well as cells within the tumor microenvironment, including macrophages and endothelial cells producing biologically relevant sources of GAS6 [93]. This allows for the development of several classes of AXL inhibitors including those that neutralize GAS6, target the AXL receptor, or inhibit AXL tyrosine kinase activity (Figure 3). Moreover, analysis of *Axl* and *Gas6* germline knockout mice shows that the GAS6/AXL signaling cascade is not required for embryonic development or normal tissue function indicating that specific GAS6/AXL inhibitors may not be associated with normal tissue toxicities [9,10].

Several classes of AXL inhibitors have been developed and have shown efficacy in preclinical models of cancer. In 2013, the first AXL inhibitor, BGB324, entered clinical trials and is currently in Phase 1b clinical trials as a single agent and in combination with cytarabine in acute myeloid leukemia. BGB324 is also being tested in combination with erlotinib in non-small cell lung cancer (BerGenBio). BGB324 was originally developed by Rigel as a small molecule TKI inhibitor with potent activity against AXL (R428, [94]). Numerous preclinical studies have effectively utilized R428 to inhibit tumor metastasis [17,65,94]. However, the biochemical selectivity for AXL over other RTKs such as Tyro3 (14-fold), Mer (16-fold), Tie2 (3-fold), Ret (9-fold), and Abl (9.3-fold) are not significant, suggesting that additional RTKs may be affected by R428 treatment [94]. There are a number of additional small molecule TKI inhibitors that are approved or in clinical trials that have reported anti-AXL activity. Among these agents, cabozantinib, a small-molecule TKI that targets VEGFR, MET, RET, cKIT, and FLT1/2/3 as well as AXL, is FDA approved for the treatment of kidney cancer and medullary thyroid cancer. A phase II trial demonstrated efficacy in renal cancer, with an objective response rate of 28%, stable disease rate of 62%, and median progression-free survival of 14.7 months [95]. While small molecule inhibitors have advantages in cost and promiscuity, it is important to balance these benefits with the risk of side effects. In particular, gene targeting studies in rats and mice have revealed that mutation or deletion of Mertk results in retinal dystrophy that is associated with a defect in pigmented retinal epithelial cells to ingest apoptotic material shed by rods and cones (recently reviewed in [84]). Moreover, Tyro3/AXL/Mertk triple knockout mice develop severe lymphoproliferative disease and autoimmunity associated with a defect in macrophage and dendritic cell clearance of apoptotic cells and inability to dampen inflammatory immune responses (recently reviewed in [84]). The risk of side effects associated with promiscuous TKIs needs to be carefully considered, especially when considering combination therapies for patients with advanced disease.

In an effort to develop more specific AXL inhibitors, a variety of biologic agents including anti-AXL antibodies and soluble AXL decoy receptors have been developed and tested in preclinical models. Notably, a soluble AXL decoy receptor-Fc fusion molecule, that has been affinity matured for enhanced GAS6 binding over the wildtype soluble AXL (80-fold enrichment), has generated specific and potent antitumor responses as a single agent therapy in preclinical models of cancer [96]. While specific biologic agents targeting AXL signaling are developed at a higher cost compared to small molecules, these agents have key advantages over small molecule inhibitors due to the high specificity and reduced risk for normal tissue toxicities that may make them more desirable for combination therapies that will be needed in the setting of advanced disease.

## 7. Concluding Remarks

AXL has emerged as a promising anti-cancer target. While AXL is rarely mutated in human cancers, the majority of tumors overexpress AXL during tumor progression and/or drug treatment to promote progression, metastasis and resistance. Genetic and pharmacologic inhibition of GAS6/AXL signaling in tumor models has begun to illuminate the diverse mechanisms by which this signaling pathway promotes tumor progression and drug resistance. However, important questions still remain in the field.

Tumor stromal crosstalk plays an important role in tumor initiation, progression, and response to therapy. Tumor cells communicate with a variety of stromal cells including fibroblasts, bone marrow derived cells, immune, vascular, and epithelial cells to facilitate tumor progression, metastasis, and therapy resistance [97]. The majority of work in the field has focused on the role of AXL signaling in tumor cells and its impact on tumor behavior. With the generation of AXL deficient mice and the use of AXL inhibitors in immune competent tumor models, we will be able to better understand how GAS6/AXL signaling is utilized in tumor stromal crosstalk to promote tumor progression [9]. Recent studies have indicated that GAS6/AXL signaling has the potential to influence the tumor vasculature, paracrine crosstalk between tumor cells and bone marrow derived stromal cells, tumor-fibroblast interactions, as well as NK cell and macrophage function [48,87,90,98,99]. Future studies investigating the role of AXL signaling within individual stromal cell populations are needed to better understand the diverse roles of GAS6/AXL signaling within the tumor microenvironment.

In addition, it will be important to further elucidate the role of GAS6/AXL signaling in mediating receptor tyrosine kinase crosstalk. The TAM family members have been shown to crosstalk and cooperate with each other and with other RTKs in the activation of downstream signaling events (reviewed in [84]). These studies may have important implications in therapeutic resistance and indicate novel combination therapies for the treatment of cancer.

Genetic and therapeutic inhibition of GAS6/AXL signaling has been utilized to identify important functional roles for AXL signaling in tumor progression, metastasis, and drug resistance. As a result, there are a variety of AXL inhibitors that are in preclinical and clinical development for the treatment of cancer. Future studies are needed to thoroughly investigate the cellular and molecular mechanisms by which AXL signaling promotes tumor progression in order to develop the most effective anti-AXL combination therapies.

## Figures and Tables

**Table 1:** AXL expression in human cancers correlates with poor prognosis, metastasis, and drug resistance.

Tumor Type	References
Acute myeloid leukemia	[30]
Breast	[28]
Colorectal	[21]
Esophageal adenocarcinoma	[17,25]
Glioblastoma multiforme	[14]
Head and neck squamous cell carcinoma	[22]
Hepatocellular carcinoma	[26]
Lung adenocarcinoma	[13,31,37]
Melanoma	[29,32,33]
Oral squamous carcinoma	[18]
Osteosarcoma	[27]
Ovarian adenocarcinoma	[20,24]
Pancreatic ductal adenocarcinoma	[15]
Pleural mesothelioma	[19]
Renal cell carcinoma	[16,34]
Urothelial carcinoma	[23]

**Table 2:** GAS6 expression in human cancers correlates with poor prognosis, metastasis and drug resistance.

Tumor Type	References
Acute myeloid leukemia	[35]
Gastric	[38]
Glioblastoma multiforme	[14]
Lung adenocarcinoma	[37]
Oral squamous carcinoma	[39]
Ovarian adenocarcinoma	[36]
Renal cell carcinoma	[16]
Urothelial carcinoma	[23]
